# Scientometric Analysis of Disaster Risk Perception: 2000–2020

**DOI:** 10.3390/ijerph182413003

**Published:** 2021-12-09

**Authors:** Tianlong Yu, Hao Yang, Xiaowei Luo, Yifeng Jiang, Xiang Wu, Jingqi Gao

**Affiliations:** 1 School of Engineering and Technology, China University of Geosciences (Beijing), Beijing 100083, China; 1002184115@cugb.edu.cn (T.Y.); wuxiang@cugb.edu.cn (X.W.); 2002190082@cugb.edu.cn (J.G.); 2 Department of Architecture and Civil Engineering, City University of Hong Kong, Hong Kong 999077, China; xiaowluo@cityu.edu.hk; 3 China Electric Power Research Institute, Beijing 100192, China; jiangyifeng@epri.sgcc.com.cn

**Keywords:** disaster risk perception, CiteSpace, web of science, visual analysis, knowledge graph

## Abstract

This paper used 1526 works from the literature on disaster risk perception from 2000 to 2020 in the Web of Science core collection database as the research subject. The CiteSpace knowledge graph analysis tool was used to visual analyze the country, author, institution, discipline distribution, keywords, and keyword clustering mapping. The paper drew the following conclusions. Firstly, disaster risk perception research has experienced three stages of steady development, undulating growth, and rapid growth. Secondly, the field of disaster risk perception was mainly concentrated in the disciplines of engineering, natural science, and management science. Thirdly, meteorological disasters, earthquakes, nuclear radiation, and epidemics were the main disasters in the field of disaster risk perception. Residents and adolescents were the main subjects of research in the field of disaster risk perception. Fourthly, research on human risk behavior and risk psychology and research on disaster risk control and emergency management were two major research hotspots in the field of disaster risk perception. Finally, the research field of disaster risk perception is constantly expanding. There is a trend from theory to application and multi-perspective combination, and future research on disaster risk perception will be presented more systematically. The conclusion can provide a reference for disaster risk perception research, as well as directions for future research.

## 1. Introduction

Risk perception is a concept used to describe people’s attitudes and intuitive judgments about risk. In a broad sense, it can also refer to people’s general assessment and reflection of risk [1]. According to Slovic, risk perception is an individual’s perception of the possible objective risks in his or her intuition about the environment, and his or her behavior of assessing the risks. Slovic argued that an individual’s subjective judgment and experience gained would affect an individual’s risk perception [2]. Sitkin, on the other hand, defined risk perception as an individual’s assessment of environmental risk, including the probability of assessment of environmental uncertainty, controllable probability, and assessment confidence [3]. He pointed out that risk perception is a decisive element of risk-taking behavior [4].

As for disaster, Fritz, a famous American disaster social scientist, has the most recognized definition by researchers. He believed that a disaster is an event with time-space characteristics that cause shocks to society or other branches of society, leading to disruptions in the social structure and disruptions in the functioning of the survival support systems of its members [5]. Subsequently, Quarantelli made a detailed discussion on the disaster in “What Is a Disaster” by combining the viewpoints of various scholars [6,7] and further refined the definition of disaster. To sum up, disaster refers to the phenomenon of loss and harm to human social life, production, life, health, and property caused by irresistible factors; it mainly refers to natural disasters (such as earthquakes, typhoons, flood, debris flow, epidemic, etc.) and man-made disasters (such as radiation, terrorism, man-made fire, traffic disasters, etc.) [6,7]. Correspondingly, disaster risk perception means the process by which the public learns the information about disaster risk and makes choices, attitudes, and behaviors to avoid disasters or reduce disaster losses according to their situation [8]. The research on disaster risk perception aims to explore how disaster risk perception will affect people’s choices, attitudes, and behaviors towards disaster prevention, so as to enable people at risk to avoid risks or accept lower risks [9].

Urbanization has accelerated in countries worldwide in the past 20 years, and the negative effects of global warming and sea-level rise have become increasingly apparent [10]. Natural disasters, such as floods, mudslides, typhoons, and earthquakes, have occurred frequently, causing huge casualties and property losses and seriously hindering the sustainable development of human society [11]. In addition, the Fukushima nuclear leak, COVID-19, and other comprehensive disasters have also aroused widespread attention worldwide, affecting people’s work and life to a certain extent [12,13]. How to systematically study disaster risk perception in order to control disaster risk has become a hot topic for scholars worldwide. In order to summarize the research status and development prospects of disaster risk perception in recent decades, Wachinger et al. reviewed the main insights related to natural disaster risk perception [14] and Ho et al. explored the impact of different disaster characteristics on risk perception [15]. Although these papers have analyzed the research characteristics of disaster risk perception, they are all based on qualitative analysis rather than quantitative analysis, and the analysis content is not comprehensive enough. Therefore, in this paper, we decided to use the scientometric method to quantitatively explore the research characteristics and development trend of disaster risk perception.

Scientometric analysis approach created by Price provided researchers with a new statistical analysis method for mass data [16]. It is widely used in the world. De Masi et al. applied it to study the connections between natural disasters and cultural heritage [17]. Olawumi et al. did a scientometric review of global research on sustainability and sustainable development [18]. Scientometric analysis explains how to uniquely search for research frontiers, that is, to study trends in related fields by using citation rules that exist in various works of the literature. This method is based on scientific methods, such as co-author analysis, journal analysis, institution analysis, co-citation analysis, and keyword analysis, and uses visual analysis technology to explore the characteristics of the development trend and research hotspots of the discipline [19]. Using scientometrics to explore the research of disaster risk perception can help to improve scholars’ understanding of the research status and significance of disaster risk perception, reveal its internal development law, and promote the sustainable development of disaster risk perception research. Therefore, we tended to use the CiteSpace visualization tool designed by Dr. Chen and his team [20] to conduct a scientometric analysis on the disaster-risk-perception literature from 2000 to 2020 included in the Web of Science Core Collection. Based on the analysis results, this paper systematically provides the overall situation of disaster risk perception research, finds the hotspots of disaster risk perception research, and puts forward constructive suggestions for the future development of disaster risk perception. The main objectives of this study were as follows: (1) to analyze the distribution and cooperation of disaster risk perception researchers and institutions, (2) to analyze the research status of disaster risk perception, and (3) to explore the research topics and hotspots of disaster risk perception.

## 2. Methods

### 2.1. Research Methods

Knowledge graph refers to an econometric method that visualizes the internal structure, disciplinary characteristics, research frontiers, and other information of a certain amount of scientific-literature information utilizing computer technology, statistics, graph theory, and other means [20,21]. A wide range of knowledge graph and science mapping tools are available for scientometric analysis, such as VOSviewer, BibExcel, CiteSpace, CoPalRed, and so on. The different tools for scientometric analysis have different capabilities and strengths [22]. Among them, CiteSpace has an outstanding effect in analyzing the trend and hotspots of discipline research [23], and this is in line with the research objective of this paper. Thus, CiteSpace was chosen as the tool for scientometric analysis in this paper.

In 2006, Dr. Chen developed the visual literature-analysis software CiteSpace and widely promoted it [24,25]. With this as the node, many scholars began to use the CiteSpace knowledge graph to analyze research hotspots in disciplines. CiteSpace generates a series of visual knowledge maps to explore the research status, research hotspots, evolution process and discipline structure of a scientific field. Based on the above analysis, researchers can master the research direction of institutions and authors, and judge the classical literature and related auxiliary research. In the field of international scientific research, CiteSpace software is widely used in computer science, information science, medicine and more than 60 fields [24]. As for the field of disaster risk perception, in 2016, Fei et al. conducted a systematic analysis on the research status of international flood risk perception by using CiteSpace software and methods, such as literature co-citation analysis, keyword co-occurrence analysis, and emergent word analysis [26]. However, the research on overall disaster risk perception in the world is still relatively blank.

In this paper, based on the Web of Science (WoS) Core Collection, the visual literature-analysis software CiteSpace (Citespace 5.7R5: Philadelphia, PA, USA) was used to analyze, excavate, and visualize data from the scientific research literature [24]. The potential knowledge and trend in the field of disaster risk perception were scientifically analyzed and revealed by the method of bibliometrics.

### 2.2. Data Source

Bibliographic databases commonly used by scholars include Google Scholar, Scopus, PubMed, and Web of Science. Among them, Web of Science (WoS) is a large multi-disciplinary core journal citation database covering engineering, natural science, social science, and other fields; the database includes many authoritative and influential academic journals in the world. At the same time, relevant studies show that the WoS database shows a better knowledge map effect when CiteSpace is used for visual analysis [27,28]. The WoS Core Collection is a multidisciplinary large-scale comprehensive citation index database based on WoS. It is a dynamic and updated digital research environment integrated by core academic information resources. It integrates high-quality information resources, analysis tools and professional software seamlessly through powerful retrieval technology and content-based connectivity, with multiple functions of knowledge retrieval, extraction, analysis, evaluation, management, and publication. Therefore, in order to effectively analyze the status and development trend of disaster risk perception, this paper chooses Web of Science Core Collection as the sample database.

The data-acquisition process was as follows. First, the Web of Science Core Collection database was selected for a basic search. We took into consideration that, in some years before 2000, such as 1998 and 1999, no articles related to disaster risk perception were published, and 2021 has not passed yet. The search time was selected between 2000 and 2020. Dr. Chen believes that when investigating a rapidly growing field, topic searches can obtain a more comprehensive dataset than keyword searches [20]. Therefore, this article chose the topic search. To ensure the comprehensiveness of the data, the search words must be chosen with great care [29]. We chose “disaster”, “risk perception”, “risk psychology”, “risk behavior”, and other searching words and tried to combine them. Finally, two retrieval words, “disaster” and “risk perception”, were selected. The word “risk perception” was used to restrict the research content, and the word “disaster” was used to restrict the research field. The search of the literature was carried out by searching for two topics at the same time. It was expressed by a search formula: “TS = risk perception * AND TS = disaster *”. The document type was set as “Article”. It means that only journal articles are retained and review articles were excluded. A total of 1568 records were obtained from the WoS database. Before the research, we conducted a detailed study on the titles, abstracts, and keywords of these records. If the literature abstract was related to disaster risk perception and related fields, then the record was retained. However, some articles also use terms related to disaster risk perception in the abstract but mainly focus on other objects, such as Hopf et al.’s study on people with communication disabilities [30]. These records were excluded. Each record needs to be carefully screened. After filtering out a few relevant records and deleting duplicate data samples in CiteSpace, we finally retained 1526 records of the literature as research samples in this paper.

## 3. Results

### 3.1. Time Distribution of Output

**Figure 1:**
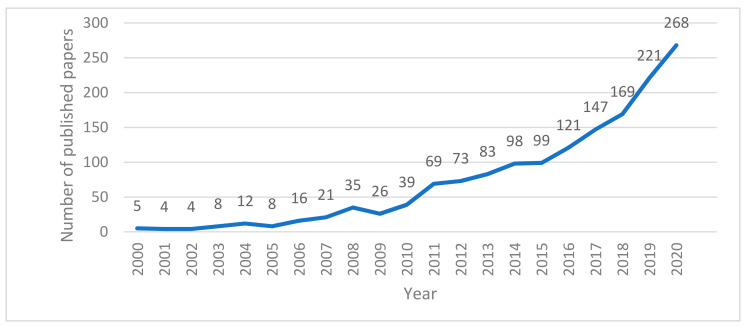
Time distribution diagram of publication volume in the field of disaster risk perception.

The time distribution of the number of published papers in the field of disaster risk perception from 2000 to 2020 is shown in Figure 1. The number of published papers on disaster risk perception research has gone through three stages: steady development, undulating growth, and rapid growth. From 2000 to 2007, the number of published papers on disaster risk perception developed steadily. In this period, the average number of published papers was 9.75, and the total number of published papers was relatively small. Disaster risk perception research was in a preliminary stage of development. From 2008 to 2010, the number of published papers on disaster risk perception fluctuated and increased, with an average annual number of 33.33. From 2011 to 2020, the number of papers published on disaster risk perception increased rapidly, with an average annual number of 134.8 and an average annual growth rate of 22.61%. On the whole, the number of research papers on disaster risk perception shows a steady growth trend and a high growth rate, indicating that disaster risk perception is gradually becoming a hot research issue in the world.

### 3.2. Characteristics of National or Regional Cooperation

**Figure 2:**
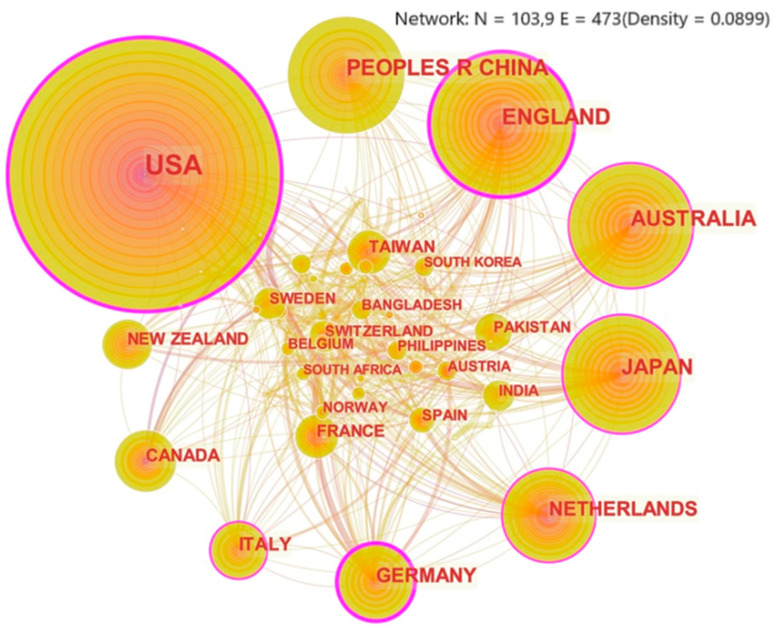
Network graph of national or regional cooperation. (Note: The size of circular nodes in the graph is proportional to the number of published papers, and the thickness of the purple circle is the size of betweenness centrality. The line between each node in the graph means that two countries or regions appear together in the literature; that is, two countries or regions are considered to have a cooperative relationship [31], and the thickness of the line reflects the strength of the relationship).

The national or regional cooperation graph reveals the distribution and intensity of cooperation between countries or regions [24]. By analyzing 1526 works of the literature, this paper obtained the national or regional cooperation graph in disaster risk perception, as shown in Figure 2.

The number of nodes in the figure is 103 and the number of links is 473, which indicates that scholars from 103 countries and regions have conducted researches on disaster risk perception and that there is a certain degree of cooperation among these countries and regions. The three countries and regions with the largest nodes are the USA, China, and England, indicating that these three countries and regions have published the most research on disaster risk perception. Table 1 shows the top 15 countries and regions by the number of publications. The results show that the top five countries and regions in terms of published papers are the USA, China, England, Australia, and Japan. Moreover, the total number of published papers by the five countries and regions accounts for 72.8% of the total number of published papers. We believe that the large number of articles published in the field of disaster risk in China and the United States is mainly due to their vast territories and frequent disasters. England, Australia, and Japan rank high mainly because of their strong awareness of disaster management. From the perspective of betweenness centrality, the betweenness centrality of the USA, England, and Germany is higher than 0.2, indicating that these three countries and regions have close cooperation with other countries and regions and have a great influence in the world. Although China, Japan, the Netherlands, and other countries have a high number of published papers, their national cooperation intensity is lower than the USA, England, and Germany. For these countries and regions, the degree of foreign exchange and international influence still needs to be strengthened.

In addition, we can see from the figure that a large part of the connecting line from Germany is wide. It shows that Germany might play a leading role in the cooperation with other countries and regions, and some European countries, such as the Netherlands and Spain, are greatly influenced by Germany in the research field of disaster risk perception. Moreover, we can see that most of the connecting lines from China are fine. It indicates that Chinese scholars prefer independent research; and, on the other hand, it indicates that China has less influence on other countries in collaborative research. The reasons for these phenomena above are probably due to the influence of culture and international relations.

### 3.3. Characteristics of Author Cooperation

**Figure 3:**
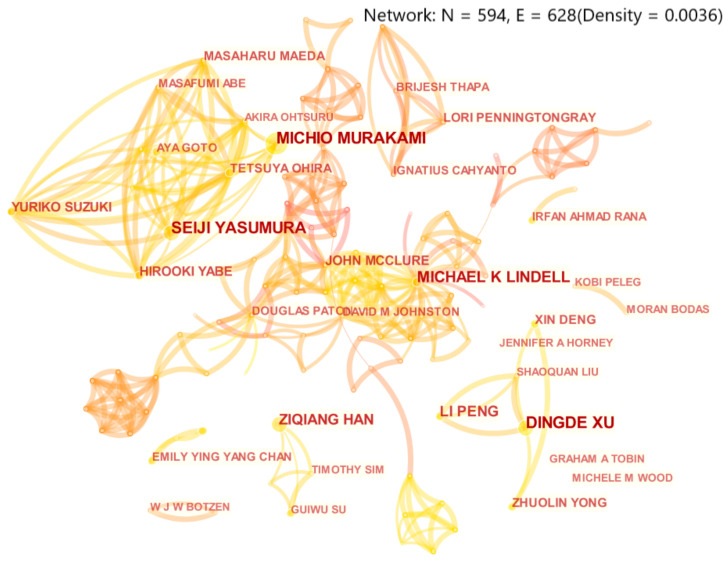
Network graph of author cooperation. (Note: The size of circular nodes in the graph reflects the number of papers published by the current author. The connection between nodes means that there are different authors in a paper simultaneously, so it is considered that there is a cooperative relationship between these authors, and the thickness of the connection indicates the strength of cooperation [24]. Set citation Counts to 5. That is, only authors with 5 or more publications are displayed).

Author collaboration networks can show the key figures in a field and the cooperative relationship between researchers [24]. As shown in Figure 3, there are a total of 594 nodes and 628 links in the figure, indicating that 594 scholars have conducted research on disaster risk perception from 2000 to 2020, and there is a certain degree of cooperation between them. In general, the research in the field of disaster risk perception was scattered as a whole but concentrated in a small part. The research team formed by Dinde Xu, Xin Deng, et al. and the research team formed by Michio Murakami, Seiji Yasumura, et al. were the most important research teams. Based on Table 2, the five authors with the most publications are Dingde Xu, Michio Murakami, Seiji Yasumura, Ziqiang Han, and Michael K. Lindell. Except for Michael K. Lindell, the starting year of these scholars was late, indicating that these scholars still entered the field of disaster risk perception in a relatively short time. At the same time, the lines in the author collaboration network are scattered, and the betweenness centrality of each scholar is low, indicating that each scholar and research team prefer independent research, and the communication and cooperation between scholars are not rich enough.

### 3.4. Characteristics of Research Institution Cooperation

**Figure 4:**
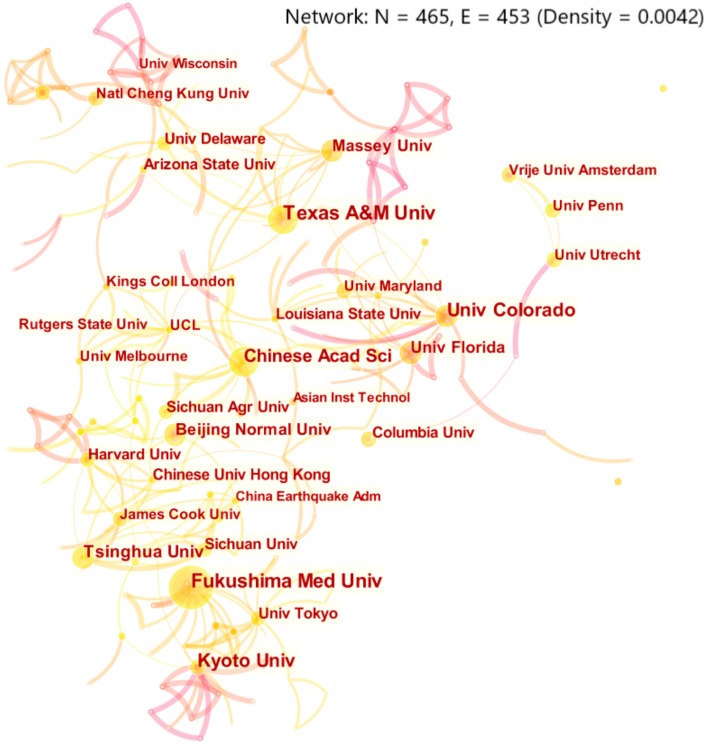
Network graph of institution cooperation. (Note: The size of circular nodes in the graph reflects the number of papers published by the institution. The connection between nodes means that different institutions appear simultaneously in a paper, so it is considered that there is a cooperative relationship between these institutions [24]. Set citation Counts to 8. That is, only organizations with eight or more publications are displayed).

The graph of institutional cooperation network can show the research centers and institutions in a field, as well as the cooperative relationships among institutions [32]. Figure 4 is the network graph of institutions obtained from this analysis, from which we can see that a total of 465 institutions have conducted researches on disaster risk perception. Texas A&M University has the largest number of published papers, with 27. Among the top 10 institutions in terms of publication volume shown in Table 3, four are in China, three are in the United States, two are in Japan, and one is in New Zealand. Institutions in China, Japan, and the United States have achieved important positions in the field of disaster risk perception research. From the perspective of betweenness centrality, Texas A&M University, Massey University, and Colorado State University have the highest betweenness centrality, indicating that these three universities have richer exchanges with other institutions and have greater influence. Fukushima Medical University mainly studied the disaster risk perception related to nuclear radiation, so it had less cooperation with institutions in different regions and its betweenness centrality was low.

### 3.5. Characteristics of Discipline Distribution

**Figure 5:**
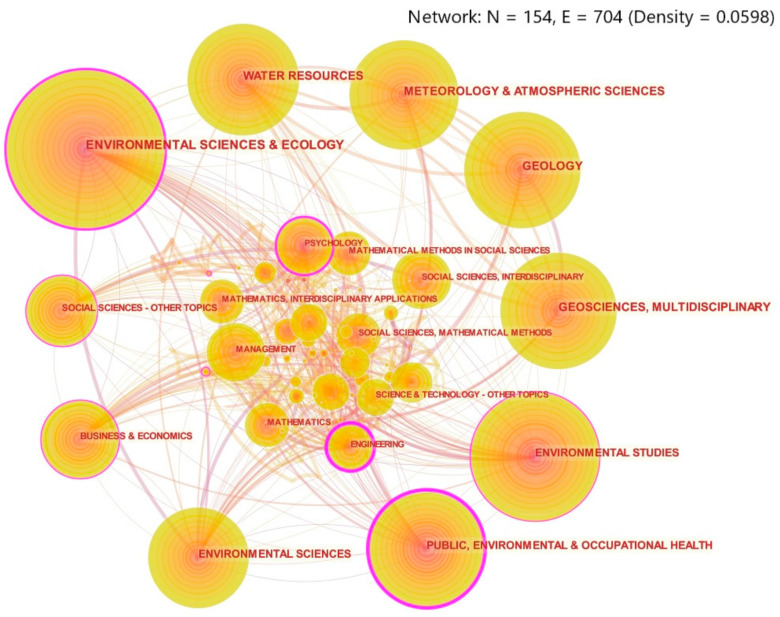
Discipline co-occurrence graph. (Note: The size of the circular node reflects the co-occurrence frequency of disciplines, and the thickness of the purple circle represents the size of betweenness centrality. The connection between nodes means that a paper belongs to different disciplines simultaneously, so these disciplines are considered to be related [33]. Set citation Counts to 54. That is, only names of disciplines with co-occurrence frequencies of 54 and above are displayed.).

As shown in Figure 5, research on disaster risk perception mainly focuses on environmental science and ecology, water resources, meteorology and atmosphere science, geology, geoscience multidisciplinary, and so on. Table 4 shows the betweenness centrality and category of Top 10 disciplines ranked by subject co-occurrence frequency. The results showed that engineering accounted for 40%, natural science 30%, management science 20%, economics 10%. Environmental science and ecology, as well as public environment and occupational health, have the most intersections. These two disciplines have the highest betweenness centrality and are closely linked with other disciplines. The betweenness centrality of meteorology and atmospheric science, geology, and geoscience multidisciplinary is 0, indicating that these disciplines are more independent and suitable for independent research. In general, the research of disaster risk perception involves a wide range of disciplines, thus requiring joint exploration by scholars from all walks of life.

### 3.6. Keyword Analysis

#### 3.6.1. Keywords Graph Analysis

**Figure 6:**
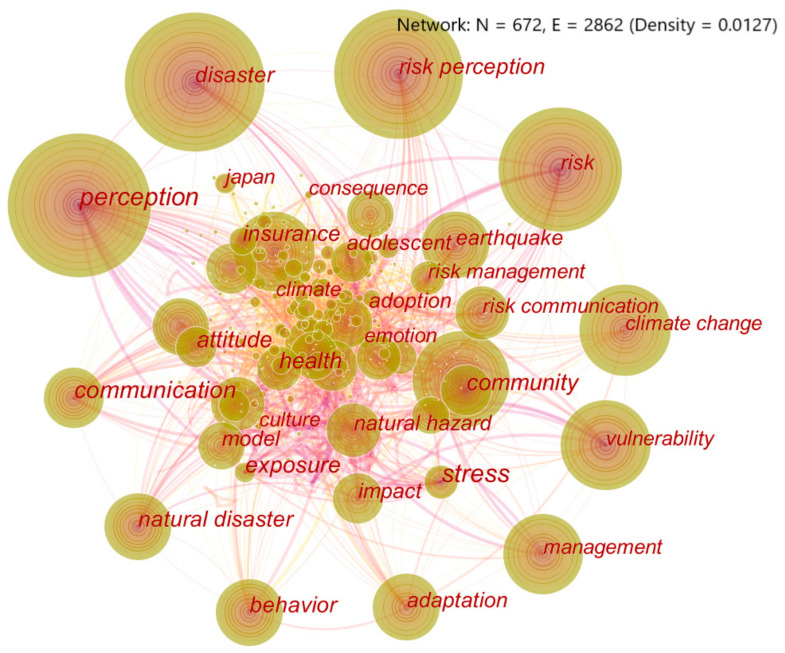
Keyword co-occurrence graph. (Note: The size of the circular node in the graph reflects the co-occurrence frequency of keywords. The connection between nodes means that different keywords appear in a paper simultaneously, so these keywords are considered to be related [30]. Set citation Counts to 60. That is, only keywords with co-occurrence frequencies of 60 or more are displayed).

Keywords are the subject and content of highly refined papers. Comprehensive analysis of keywords in the paper shows that, the higher the co-occurrence frequency, the more related topics and contents are involved. Thus, keywords can be used to determine hot issues in a research field [34]. In Figure 6, there are 672 nodes and 2862 connections. The five nodes with the highest frequency are “perception” (462 times), “disaster” (453 times), “risk perception” (431 times), “risk” (365 times), and “climate change” (258 times). The five nodes with the highest betweenness centrality are “perception” (0.14), “disaster” (0.08), “risk” (0.06), “earthquake” (0.06), and “communication” (0.06). After a comprehensive analysis, “perception”, “disaster”, “risk”, “climate change”, and “earthquake” were the top five hot keywords in the research field.

**Figure 7:**
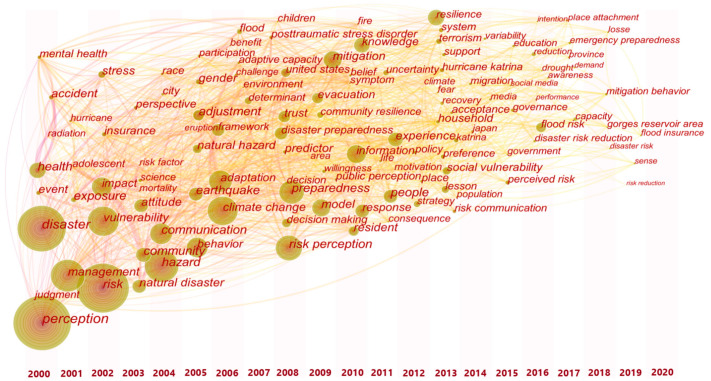
Time-zone view of keyword co-occurrence network.

In order to further summarize hot keywords, the keyword co-occurrence network was displayed in the time-zone view in Figure 7. It shows that the number of topics has increased significantly from 2004 to 2014. The hot keywords mentioned above all appeared in an earlier time period. Among the new keywords appearing in the last five years, “flood risk” has a high-risk frequency, which may be closely related to the practical factors of the frequent storm and flood disasters around the world in recent years. In addition, keywords around risk control and emergency management, such as “disaster risk reduction”, and “emergency preparedness”, have also appeared more frequently in recent years.

#### 3.6.2. Keywords Cluster Analysis

**Figure 8:**
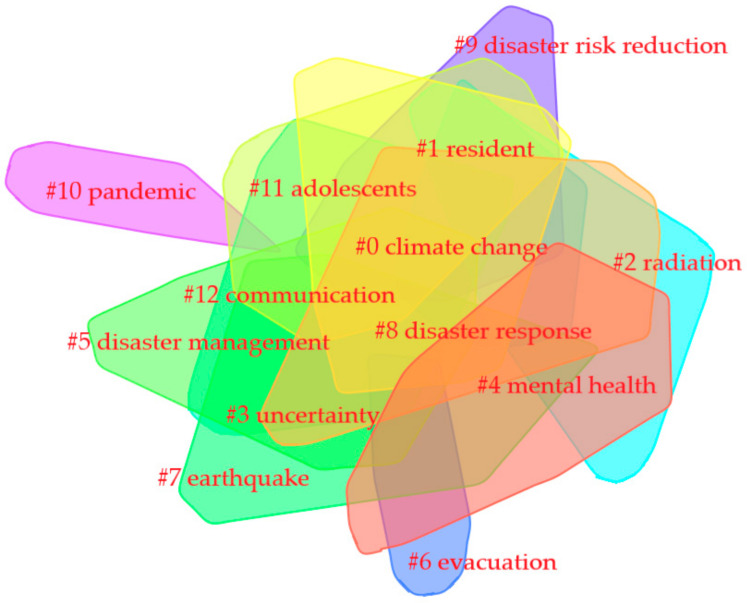
Keyword clustering map.

To find the relationship between high-frequency keywords and further reveal the research hotspots in the field of disaster risk perception, this study classified high-frequency keywords through high-citation cluster analysis of disaster risk perception [35]. It drew the correlation clustering map in Figure 8. The 13 clusters in the figure have mean silhouette values greater than 0.8, indicating good homogeneity. The analysis was reliable.

As shown in Table 5, the 13 representative clusters are #0Climate Change, #1Resident, #2Radiation, #3Uncertainty, #4Mental Health, and #5Risk Management, #6Evacuation, #7Earthquake, #8Disaster Response, #9Disaster Risk Reduction, #10Pandemic, #11Adolescents, and #12Communication. The 13 clusters are mainly divided into four themes, which are research disaster categories (including tags #0, #2, #7, and #10), risk perception survey object (including tags #1 and #11), risk behavior and risk psychology research (including tags #3, #4, #8, and #12), and risk control and emergency management research for disasters (including tags #5, #6, and #9).

#### 3.6.3. Emergent Keyword Analysis

**Figure 9:**
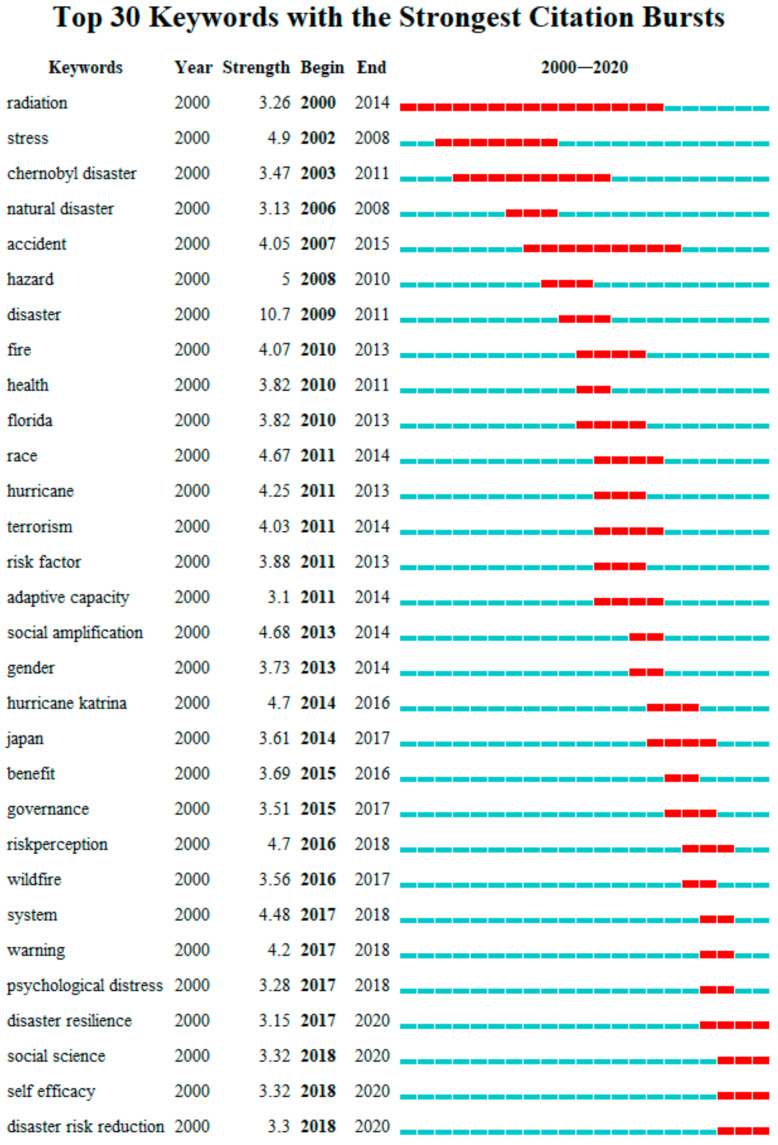
Emergent keyword map.

Emergent keywords refer to the keywords that appear many times in a certain period. Based on the time trend of keywords, we can predict the dynamic research hotspots in a certain field [36]. The map of emergent keywords obtained in this analysis is shown in Figure 9. A total of 30 emergent keywords are obtained. According to the emergence time of keywords, we can divide the period from 2000 to 2020 into three stages. The period from 2000 to 2009 was the first stage, in which keywords such as “radiation”, “stress”, “Chernobyl disaster”, and “natural disaster” appeared, indicating that the focus of attention in this period was on the relationship between disaster itself and risk perception. Moreover, nuclear disaster as a hot disaster has been widely studied. The second stage was from 2010 to 2014, with emergent keywords such as “fire”, “hurricane”, “terrorism”, “adaptive capacity”, and “social amplification”. The types of disasters in this period tended to be diversified. The focus shifted to the relationship between social factors, individual factors, and disaster risk perception. The third stage was from 2015 to 2020, and the emergent keywords were “governance”, “warning”, “social science”, “self-efficacy”, “disaster resilience”, etc. It indicated that the social and individual factors of disaster risk perception in this period were further expanded and deepened, and the focus gradually shifted to risk control and emergency management.

In general, emergent keywords can reveal social reality, as well as research trends, in disaster risk perception to a certain extent. For example, the word “terrorism” came up frequently from 2011 to 2014. The main reason is that there were a number of serious terrorist attacks around the world during this period. In terms of research trends, the frequent emergence of words such as “disaster resilience”, “disaster risk reduction”, “social science”, and “self-efficacy” after 2017 may also indicate that the research in the field of disaster risk perception will become practical and comprehensive.

## 4. Discussion

### 4.1. Research Hotspots of Disaster Risk Perception

**Figure 10:**
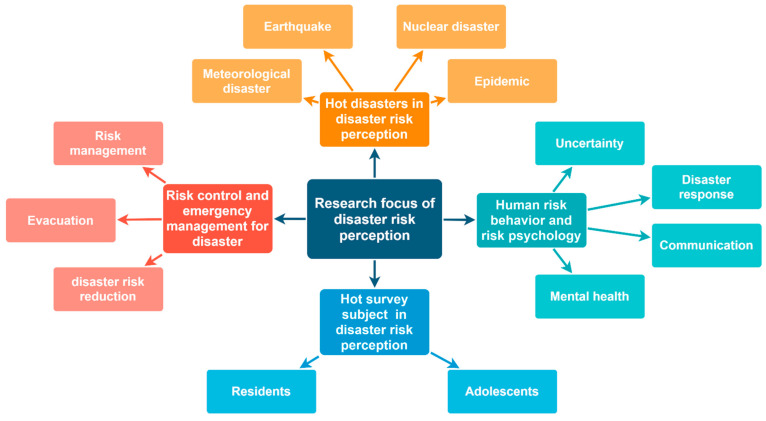
Research hotspots of disaster risk perception.

Through the analysis of keywords, research hotspots in the field of disaster risk perception are summarized in Figure 10.

According to keyword graph analysis and keyword cluster analysis, we can get research hotspots in the field of disaster risk perception. First of all, in terms of disaster categories, “climate change” and “earthquake” were the top five hot keywords obtained from the comprehensive analysis of the graph. It indicated that meteorological disasters and earthquake disasters, as two traditional natural disasters, have always been the hot issues studied by scholars in the field of disaster risk perception. Taking meteorological disasters as an example, the global climate pattern has changed dramatically in the past 20 years, and the impact of extreme weather events, such as droughts, heavy rains, and hurricanes, on global ecosystems has received much attention from scholars worldwide. Grothmann’s and Botzen’s research among them is the most classic. Grothmann et al. proposed a social psychological model for flood disasters based on the protection motivation theory. They used residents’ views on previous flood experiences and future flood risks to infer the reliability of social public flood control, the effectiveness and cost of residents’ self-protection behavior, and residents’ perception of flood risk. This model effectively guides public risk communication and is of great significance to flood risk management [37]. Botzen et al. investigated about 1000 homeowners in the Netherlands. They used a variety of models to estimate the impact of socioeconomic and geographical characteristics, personal flood experience, flood threat knowledge, and personal risk attitude on the formation of risk perception. They pointed out that the difference in expected flood risk is always related to the actual risk level [38]. Due to scholars’ emphasis on meteorological disasters and earthquake disasters, the research methods of risk perception in these areas were rich, and the research content was relatively mature and specific.

In addition, besides traditional natural disasters, “#2 radiation” and “#10 epidemic” also appeared in the keyword clustering map as comprehensive disasters. These disasters have a wider impact. They are more likely to trigger social panic, and often attract the attention of a large number of scholars in a certain period. Since the Fukushima nuclear accident in 2011, relevant scholars have performed many studies on the perception of nuclear radiation risk. For example, Kunii et al. measured the mental health status of 73,569 people aged 15 and above living in the evacuation area of Fukushima Prefecture. They found that the nuclear accident had a serious impact on the mental health of residents, and the excessive perception of radiation risk greatly aggravates people’s psychological pressure [39]. Yoshida et al. conducted a questionnaire survey on 287 students from the department of nursing at a National University in Japan to study the knowledge and risk perception of radiation among nurses and doctors. The survey results showed that Japanese nursing students had little knowledge of radiation and had a great fear of X-rays. They believed that receiving appropriate radiation knowledge education can effectively reduce risk perception and thus reduce fear [40]. Following the outbreak of COVID-19 in late 2019, papers on COVID-19-related risk perception also exploded. Cvetković et al. conducted a study on the risk perception of 975 Serbian citizens during the outbreak. The survey results showed significant differences in public perceptions of the risks posed by the threat of infectious diseases, such as COVID-19. Cvetković et al. argued that emergency management agencies should use these differences to develop targeted strategies, and then strengthen community and national preparedness by promoting behavioral change and improving risk management decisions [41]. Lee et al. explored the effectiveness of obtaining information through emergency warning SMS in the early stage of the COVID-19 outbreak in South Korea and its impact on individual prevention behavior. They believe that the government should actively consider sending emergency warning messages to provide accurate and reliable information to the public, which is conducive to reducing the impact of negative news [42].

The types of disasters studied in the field of disaster risk perception are closely related to the types of disasters occurring in the current reality. Generally speaking, when a new disaster appears, scholars will conduct a lot of research on it, in order to reduce disaster losses in a short time and meet social needs. The research on risk perception of these disaster areas will also become a hot issue during this period. In addition, regional disasters, such as Fukushima nuclear radiation in Japan and hurricane disasters in the United States, are often studied in depth by local scholars. Moreover, global disasters, such as global warming and COVID-19, are often studied by scholars from all over the world. Especially for COVID-19, its emergence and rapid spread at the end of 2019 led to a proliferation of articles about it in 2020. The annual number of articles in disaster risk perception reached a new peak in 2020.

From the perspective of risk perception survey objects, “#1 Resident” and “#11 Adolescents” were the two main survey objects for disaster risk perception. Risk-perception questionnaires were usually targeted at residents or adolescents in disaster-affected areas. For example, Martins studied household disaster preparedness before Storm Sandy among 2001 residents of all five boroughs of New York [43]. When studying the role of behavioral experience in risk judgment, Halpern investigated 577 adolescents and young adults aged from 10 to 30 years old [44]. The main reason was that residents, as direct audiences of disasters, were more representative. Meanwhile, teenagers were affected by many factors such as age and education level, and the survey results varied greatly. These survey objects will be helpful for scholars to study the impact of risk perception from different perspectives.

The clusters of “#3 uncertainty”, “#4 mental health”, ”#8 disaster response”, and “#12 communication” indicated that the study of human risk behavior and risk psychology was a hot research topic in the field of disaster risk perception. In 1960, Bauer applied risk perception to consumer research and proposed the theory of uncertain consequences [45]. The word “uncertainty” firstly appeared in the field of risk perception. Later, the psychometric approach proposed by Slovic [46], the sociocultural theory proposed by Douglas and Wildavsky [47], and other theoretical paradigms had been widely used by many scholars around the world. These theoretical paradigms interpret risk behavior and risk psychology from many different perspectives, such as psychology, social science, and geography. They also interpreted and measured the public’s perception of risk under disasters. For example, McDermott used the Strengths and Difficulties Questionnaire (SDQ) to identify children and adolescents who might need psychological intervention after exposure to wildfire. He pointed out that younger individuals are more likely to appear in developmental and psychological disorders if exposed to threats [48]. This is a typical research process of the psychometric approach; that is, questionnaire data are used as the framework, and influencing factors are quantitatively analyzed by the algorithm. Jennifer et al. surveyed residents of communities downstream from glacial lakes. They found that the persistent attachment of community residents to their valleys and their desire for cultural continuity in the face of social, economic, and environmental changes significantly influenced their perception of risk [49]. This study further demonstrated the profound influence of sociocultural factors on risk perception.

The clusters of “#5 risk management”, “#6 evacuation”, and “#9 disaster risk reduction” indicated that risk control and emergency management for disasters were also hotspots in the field of disaster risk perception. The core of this part was to apply theoretical analysis and results into practice, in order to reduce disaster risk, casualties, and property losses. For example, Wallace interviewed 205 households in North Carolina to assess the connection between perceived risk and actual risk between flood and evacuation. He believed that actual flood risk was an important environmental clue for evaluating risk perception and evacuation decision-making [50]. Chatfield investigated disaster-risk communications during the eruption of Mount Sinabung in Indonesia. Based on his analysis of the Sinabung hashtag on Twitter, he found a lack of engagement in risk perception communication and leadership by the government to respond to the Indonesian public’s concerns about the Mount Sinabung disaster. In addition, Chatfield proposed that Twitter can be effectively used as a multidirectional risk communication tool to share risk perception and disaster information with the public quickly and effectively [51]. In general, this part of disaster risk perception research was more realistic and provided practical guidance in disaster risk reduction

### 4.2. Research Trends of Disaster Risk Perception

**Figure 11:**
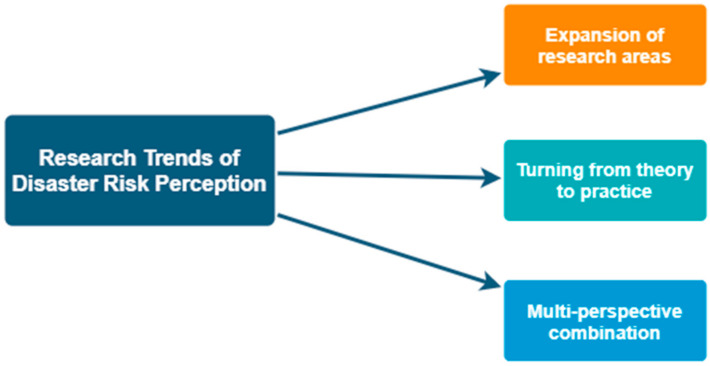
Research trends of disaster risk perception.

Through the analysis of keywords, research trends in the field of disaster risk perception are summarized in Figure 11.

According to the emergent keywords map, we can summarize the frontier trends of disaster risk perception research. On the whole, the research fields of disaster risk perception were constantly expanding. For example, in terms of the definition of disaster, the main research contents before 2009 were natural disasters and nuclear accidents, while the “terrorism” occurring from 2010 to 2014 and the “wildfire” occurring from 2015 to 2020 have expanded the disaster fields of risk perception.

On the other hand, the research on disaster risk perception tends to shift from theory to application. From the analysis results, we can see that the keywords before 2014 mostly focus on the public’s risk perception and mental health under the background of disasters. However, keywords after 2014, such as “governance” and “disaster resilience”, focus on studies of disaster risk management and emergency management, which can provide more practical guidance for public management and have more practical significance.

In addition, there is a trend of keyword development from multiple perspectives. Many factors are affecting the human cognitive process, and the factors of social and cultural background cannot be ignored [52]. Keywords such as “social science”, “health”, and “self-efficacy” indicate that future studies on disaster risk perception will comprehensively analyze the public’s risk perception of disasters from multiple perspectives, such as society, individual, and culture. The research content of disaster risk perception will be more detailed and scientific.

In general, the field of disaster risk perception in the future will be standardized and systematic. Scholars will conduct research on disaster risk perception from many different perspectives, such as psychology, behavioral science, and geology. They will also innovate research paradigms in the field of disaster risk perception. The two current research hotspots, the research on human risk behavior and risk psychology, as well as the research on disaster risk control and emergency management, will continue to be deepened. In addition, the research content in the field of future disaster risk perception will also be affected by the types of disasters that occur in the future.

### 4.3. Knowledge Gaps

Even though our understanding of disaster risk perception deepens over time, it is still difficult to apply disaster risk perception to disaster risk reduction in reality. Bubeck mentioned in his paper that the current focus on risk perceptions as a means to explain and promote private flood mitigation behavior is not supported on either theoretical or empirical grounds [53]. How to use risk perception to effectively control disaster risk is not only the current research hotspot but also the current knowledge gap. We believe that some advanced countries or institutions will devote themselves to the practical application of disaster risk perception, while some backward countries or institutions will continue to improve their theoretical systems. The continuous exploration of theory and experience can make this knowledge gap fully filled, thereby making a substantial contribution to disaster risk reduction.

### 4.4. Limitation of Research

This study used only data from the WoS Core Collection as the data source. Moreover, the research results were limited by the influence of the database. It was not convincing enough to predict the future development trend of disaster risk perception. On the other hand, this paper used CiteSpace to study disaster risk perception from multiple perspectives, including author, region, discipline, key words, and other aspects, but the pertinence was somewhat weak.

## 5. Conclusions

The in-depth study of disaster risk perception is an important measure to control disaster risk. Disaster risk can be reduced by adjusting public risk psychology and risk behavior. Therefore, using the scientometrics analysis method to explore the research work of disaster risk perception will be conducive to the development of current research, and it will effectively promote the sustainable development of disaster risk perception and related research.

This paper indicated that the research on human risk behavior and risk psychology and the research on disaster risk control and emergency management are two hotspots that have appeared in recent years. It can provide research directions for potential readers, which will push the development of disaster risk perception research so as to adjust public risk psychology and risk behavior and improve public safety.

In addition, the research field of disaster risk perception is constantly expanding. There is the trend of shifting from theory to application and the trend of combining multiple perspectives. This paper predicts that, in the future, the research on disaster risk perception will be more detailed, more specific, and more comprehensive. Scholars should focus on research hotspots such as risk behavior, risk psychology, and risk management and control. They must strengthen theory, focus on practice, and establish a perfect research system and paradigm of disaster risk perception to solve the situation of frequent disasters around the world in recent years.

## Figures and Tables

**Table 1:** Country or region cooperation features and number of published papers from 2000 to 2020.

Number of Papers	Betweenness Centrality	Country or Region	Starting Year
521	0.38	USA	2000
176	0.07	Peoples R China	2011
150	0.34	England	2002
138	0.17	Australia	2001
126	0.14	Japan	2004
79	0.14	Netherland	2000
75	0.30	Germany	2005
56	0.13	Italy	2003
52	0.07	Canada	2003
38	0.04	New Zealand	2008
38	0.01	Taiwan	2008
34	0.01	France	2003
32	0.04	Spain	2006
30	0.00	Pakistan	2012
28	0.01	Sweden	2006

**Table 2:** Top 5 authors in the number of articles published.

Name	Number of Published Papers	Starting Year
Dingde Xu	13	2017
Michio Murakami	13	2017
Seiji Yasumura	11	2016
Ziqiang Han	9	2017
Michael K. Lindell	9	2008

**Table 3:** Top 10 research institutions in the number of articles published.

Published Institution	Number of Published Paper	Betweenness Centrality	Country	Starting Year
Texas A&M University	27	0.09	USA	2005
Fukushima Med University	26	0.02	Japan	2012
Kyoto University	23	0.08	Japan	2004
Colorado State University	22	0.10	USA	2004
Chinese Academy of Sciences	17	0.02	China	2011
Tsinghua University	15	0.06	China	2017
Massey University	14	0.09	New Zealand	2014
Beijing Normal University	13	0.02	China	2011
University of Florida	13	0.01	USA	2017
The Chinese University of Hong Kong	12	0.07	China	2014

**Table 4:** Statistics of top 10 disciplines with co-occurrence frequency.

Discipline Area	Frequency	Betweenness Centrality	Discipline Category
Environmental Science and Ecology	491	0.34	Engineering
Water Resources	387	0.03	Engineering
Meteorology and Atmosphere Science	380	0	Natural Science
Geology	370	0	Natural Science
Geoscience Multidisciplinary	315	0	Natural Science
Environmental Studies	271	0.13	Engineering
Public Environmental and Occupational Health	248	0.42	Management Science
Environmental Science	146	0.08	Engineering
Business and Economics	119	0.17	Economics
Social Science/Other Topics	121	0.17	Management Science

**Table 5:** Statistics of top 10 disciplines with co-occurrence frequency.

Cluster ID	Cluster Name	Silhouette	Contain the Keywords
#0	Climate Change	0.933	Household Risk *, Adaptation *, Storm, Flooding *, etc.
#1	Resident	0.983	Environmental Concerns, Emergency Management *, Natural Disasters *, etc.
#2	Radiation	0.856	Fukushima, Causal Attribution, Government Control Level *, etc.
#3	Uncertainty	1.000	Subjectivity, Decision Making, Near Misses, etc.
#4	Mental Health	0.932	Spatial Isolation, Mobile Phone Data *, Knowledge Gap *, etc.
#5	Risk Management	0.935	Doctors And Nurses, Humanitarian Crises, Media Exposure *, etc.
#6	Evacuation	0.823	Conversation *, Risk Assessment *, Earthquake Vulnerability *, etc.
#7	Earthquake	0.877	Disaster Prevention Education *, Public Risk Perception *, Vulnerability *, etc.
#8	Disaster Response	0.823	Humanitarian Assistance *, Critical Infrastructure, Institutions, etc.
#9	Disaster Risk Reduction	0.956	Bioterrorism, Cold Weather Warnings, Income Inequality, etc.
#10	Pandemic	0.811	COVID-19 *, International Public Health Emergencies, Government Assistance, etc.
#11	Adolescents	0.896	SDQ *, Health Self-Assessment, Performance Experience *, etc.
#12	Communication	0.902	Regression Analysis, Tokai floods *, Hispanics *, etc.

*: It means that the keyword appears in multiple clusters and is shared by multiple clusters.

## Data Availability

The data presented in this study are available in the Web of Science core database.
